# Supplementary material for: Small-scale utilitarianism: High acceptance of utilitarian solutions to Trolley Problems among a horticultural population in Nicaragua
Source: PLoS One. 2021 Apr 5;16(4):e0249345. doi: 10.1371/journal.pone.0249345 (PMC8021155; doi:10.1371/journal.pone.0249345)
Supplement: S1 File — (DOCX) [file pone.0249345.s001.docx]

**Supporting Information**

**Table of Contents**

Test of survey round on likelihood to respond

with a utilitarian decision in Study 1: 2

Test of effect of order in which a vignette was

presented on rating of utilitarian decision in Study 2. 4

Study 1 Materials 6

Study 2 Materials 13

**Study 1 Order Effects**

**Table S1**. Multilevel probit analysis of the decision to act as a function of vignette and data collection round. Vignette A and round 1 serve as the reference levels. A subject identifier is included as a varying intercept to account for heterogeneity in propensities toward utilitarianism among the sampled participants. The model was estimated in R using the *brms* package. Reported parameter estimates are the posterior means and standard deviations.

| Parameter | Estimate (SD) |
| --- | --- |
| Intercept | 2.53 (0.78) |
| Round 2 | −1.14 (0.45) |
| Trolley Switch | −0.94 (0.55) |
| Trolley Push | 0.55 (0.63) |
| Stampede Gate | −1.23 (0.56) |
| Subject ID variance | 0.88 (1.27) |

Explanations for this decline are necessarily speculative, but potentially relate to other aspects of the unrelated interviews that were conducted prior to the Trolley study. That is, there were two rounds of data collection in part due to a clerical mistake that necessitated a repeat of one component of these interviews. Most participants were aware of this error, though not in detail, and they may have inferred that they were being encouraged to change their responses, including to the Trolley scenarios in this study.

**Table S2**. Frequencies of decisions to Trolley Problems when limited to only participants’ first scenario.

|  | Choosing Utilitarian | 95% Clopper-Pearson CI | Exact Test |
| --- | --- | --- | --- |
| Trolley Switch | 22/23 | (0.781 – 0.999) | p=0.097 |
| Trolley Push | 18/24 | (0.533 – 0.902) |  |
| Stampede Gate | 26/27 | (0.810 – 0.999) | p=0.015 |
| Stampede Push | 14/21 | (0.430 – 0.854) |  |

Wiegman et al. (1) found that Trolley Problem order effects were consistently reported when positively rated scenarios (e.g., the Trolley Switch and Stampede Gate) were preceded by more negatively rated scenarios (e.g., Trolley Push and Stampede Push), but not the reverse. However, here we find no such differences in order effects, although the sample sizes are quite limited once they are broken down in this way.

**Table S3a and S3b**. Frequencies of decisions to Trolley Problems for individuals who took part in both rounds of the Study 1 depending on the order of scenarios presented.

S3a. Gate/Switch Scenarios

|  | Act | Don’t Act |
| --- | --- | --- |
| Presented First | 4 (18%) | 18 (82%) |
| Presented Second, Following Push Scenario | 2 (18%) | 9 (82%) |

Fisher’s exact, p=1.00

S3b. Push Scenarios

|  | Act | Don’t Act |
| --- | --- | --- |
| Presented First | 0 (11%) | 24 (100%) |
| Presented Second, Following Switch/Gate Scenario | 1 (11%) | 8 (89%) |

Fisher’s exact, p=0.273

**Study 2 Order Effects**

**Table S4a**. Response means by scenario and order shown.

|  | Order | | | |
| --- | --- | --- | --- | --- |
|  | 1 | 2 | 3 | 4 |
| Trolley Switch | 3.73 | 3.88 | 3.86 | 3.33 |
| Trolley Push | 3.41 | 3.30 | 3.92 | 3.85 |
| Stampede Gate | 3.50 | 3.62 | 3.77 | 3.86 |
| Stampede Push | 3.32 | 3.92 | 3.18 | 3.42 |
| Truck Silent | 3.33 | 3.53 | 3.77 | 3.46 |
| Truck Call | 3.18 | 3.54 | 3.76 | 4.00 |
| Stampede Silent | 3.55 | 3.56 | 3.47 | 3.58 |
| Stampede Call | 3.91 | 3.43 | 3.53 | 3.89 |
| Overall Average | **3.46** | **3.61** | **3.66** | **3.67** |

**Table S4b**. Response n’s by scenario and order shown.

|  | Order | | | |
| --- | --- | --- | --- | --- |
|  | 1 | 2 | 3 | 4 |
| Trolley Switch | 15 | 17 | 14 | 15 |
| Trolley Push | 17 | 10 | 12 | 20 |
| Stampede Gate | 12 | 21 | 13 | 14 |
| Stampede Push | 19 | 12 | 11 | 19 |
| Truck Silent | 18 | 15 | 13 | 13 |
| Truck Call | 17 | 13 | 21 | 9 |
| Stampede Silent | 11 | 18 | 19 | 12 |
| Stampede Call | 11 | 14 | 17 | 18 |
| Overall Average | **15** | **15** | **15** | **15** |

**Table S4c**. Mixed-effects linear model exploring effect of scenario being in the first position with Scenario as a fixed effect and individual as a random effect.

|  | B | St. Err | p |
| --- | --- | --- | --- |
| Intercept | 3.780 | 0.113 | <0.001 |
| Scenario in First Position | -0.174 | 0.073 | 0.018 |
| Trolley Switch | - | - | - |
| Trolley Push | -0.119 | 0.141 | 0.397 |
| Stampede Gate | -0.050 | 0.134 | 0.709 |
| Stampede Push | -0.292 | 0.132 | 0.027 |
| Truck Silent | -0.178 | 0.135 | 0.187 |
| Truck Call | -0.189 | 0.132 | 0.153 |
| Stampede Silent | -0.204 | 0.134 | 0.128 |
| Stampede Call | -0.076 | 0.132 | 0.568 |

**Table S4d**. Comparisons of the Acceptability of Utilitarian Decisions across Trolley Scenarios using Only the First Round

|  | n | Mean | 95%CI | p-value |
| --- | --- | --- | --- | --- |
| Trolley Switch | 15 | 3.73 | 3.20-4.27 | 0.362 |
| Trolley Push | 17 | 3.41 | 2.90-3.93 |  |
| Stampede Gate | 12 | 3.50 | 2.86-4.14 | 0.660 |
| Stampede Push | 19 | 3.32 | 2.69-3.94 |  |
| Truck Silent | 18 | 3.33 | 2.79-3.87 | 0.679 |
| Truck Call | 17 | 3.18 | 2.59-3.76 |  |
| Stampede Silent | 11 | 3.55 | 2.79-4.30 | 0.377 |
| Stampede Call | 11 | 3.91 | 3.44-4.38 |  |

**Table S4e**. Estimated marginal means for acceptability of scenarios resulting from mixed-effects linear model which included whether the scenario was in the first position as a fixed effect and individual as a random effect.

|  | Estimated Marginal Mean | St. Err | p-value |
| --- | --- | --- | --- |
| Trolley Switch | 3.69 | 0.113 | 0.397 |
| Trolley Push | 3.57 | 0.114 |  |
| Stampede Gate | 3.64 | 0.115 | 0.085 |
| Stampede Push | 3.40 | 0.112 |  |
| Truck Silent | 3.51 | 0.114 | 0.940 |
| Truck Call | 3.50 | 0.113 |  |
| Stampede Silent | 3.49 | 0.114 | 0.362 |
| Stampede Call | 3.62 | 0.114 |  |

**Study 1 Scenarios**

Trolley Switch (No Contact / No Means)


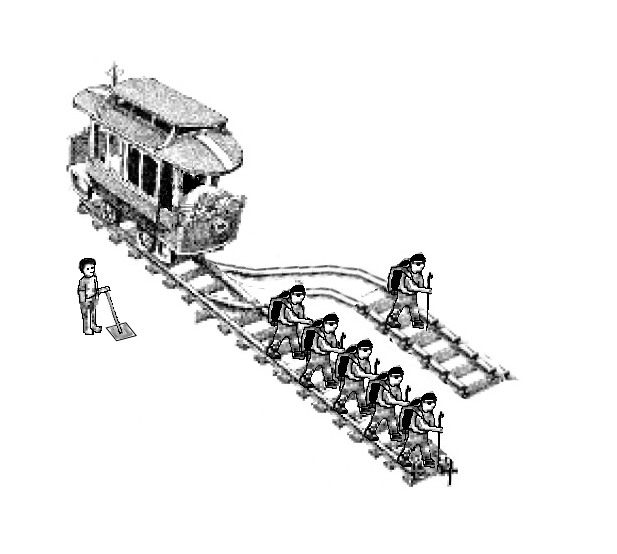


English

An out-of-control train traveling at full speed is headed for five individuals who are on the track and who will die if the train continues its current course. You are standing next to a lever that can change the train to another track if you activate it. On this track, there is only one person who will die if you activate the lever.

Would you activate the lever or let the train continue its current course?

Spanish

Un tranvía fuera de control se dirige a toda velocidad hacia cinco personas que se encuentran en la vía, las cuales morirán si el tranvía sigue su curso actual. Usted está de pie junto a un mecanismo que encaminará al tranvía por una vía diferente si lo acciona. En esa vía se encuentra una sola persona, la cual morirá si acciona el mecanismo.

¿Accionaría el mecanismo o deja el tranvía seguir su curso actual?

Mayangna

Tren as brik diska kaiwa sakki, ahahl singka tani pas yakat wicua tuluky an yaka balna dadauwa rangky tren ni aka ban kiw kat. Muih al as yaklauwi talawa witky tren ni akat pana lakwak kat tau k kau kiunini yulni, yaka tani kau al as witky an yaka dauwarangky kaput yamwa kat.

Panini aka lakma sah u datang ban tan ikat kiwarang yah tren ni aka?

Trolley Push (Contact / Means)


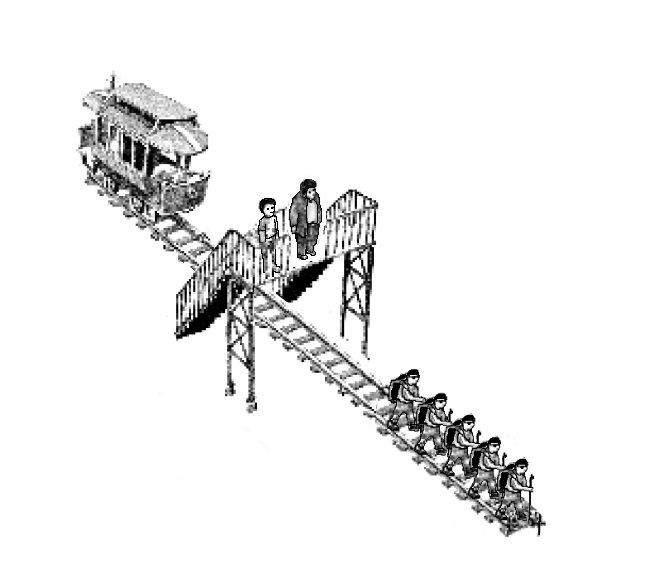


English

An out-of-control train traveling at full speed is headed for five individuals who are on the track and who will die if the train continues its current course. You are standing next to a large man on a foot bridge over the track. The only way to keep the five men from not dying is to push this man from the foot bridge into the path of the train. In this case, the large man would die but not the five individuals.

Would you push the man or let the train continue its current course?

Spanish

Un tranvía fuera de control se dirige a toda velocidad hacia cinco personas que se encuentran en la vía, las cuales morirán si el tranvía sigue su curso actual. Usted está de pie junto a un hombre grande en un puente peatonal que está sobre la vía. La única manera de salvar a las cinco personas es empujar a este hombre del puente peatonal, dentro de la trayectoria del tranvía. En este caso, el hombre grande morirá pero no las cinco personas.

¿Empujaría al hombre o deja el tranvía seguir su curso actual?

Mayangna

Tren as brik diska kaiwa sakki, ahahl singka dadauwa rangky, tren ni aka ban tan ikat kiwa kat. Muih al as witky an yaihnit kau muih al uk kas nuhni witky dak bayaknini as kau. Tren ni aka laih watnini kat di aslah yamni ki, al nuhni yahnit kau wit akat tuknini kalahwi ahahl singka daunini awasa yulni, kaput laih alni nuhni aka dau warangky. Alni aka kulwi talawi dawi tuk awarangky al aslah daunini yulni singka awasa.

Alni nuhni akat tukwarang yah u dawak kat tanit kau kiwarang yah tren ni aka?

Stampede Gate (No Contact / No Means)


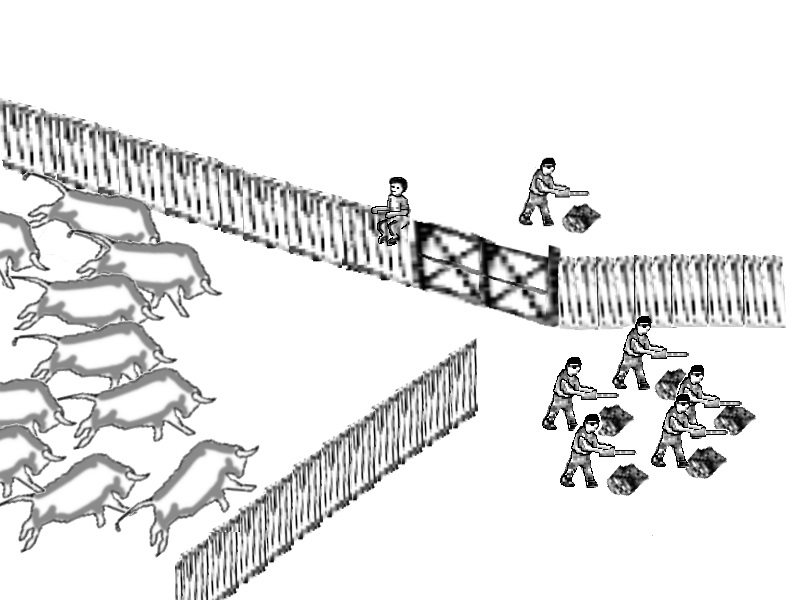


English

You are travelling far away from your community and walking along a fence. You hear a stampede and jump onto the fence. You see that the cattle are headed for an opening in the fence. Beyond this opening is five loggers cutting lumber who will die if the stampede continues its course. They do not know of the stampede due to the noise of the chainsaws and cannot hear the man’s warnings. You see that there is a gate that is to your side, and you can push it to close the opening. But if you push the gate, another opening will appear on the other side and beyond that opening is a single logger who will die if the stampede continues that course.

Would you push the gate or let the stampede continue its original course?

Spanish

Usted está viajando lejos de su comunidad y caminando a lo largo de una cerca. Oye una estampida y salta en la cerca. Usted ve que las vacas se dirigen a una abertura en la cerca y más allá de esa hay cinco motosierristas cortando madera, los cuales morirán si la estampida sigue su curso actual. Ellos no saben de la estampida por el ruido de sus motosierras, y además, no pueden oír sus avisos. Ve que hay una puerta que está al lado de usted y puede empujarla y cerrar la abertura. Pero si empuja la puerta, otra abertura se abrirá en el otro lado y más allá de esa hay un motosierrista solo, el cual morirá si la estampida sigue ese curso.

¿Empujaría la puerta o deja la estampida seguir su curso actual?

Mayangna

Muih al as tawan ni kaupak kalahna likky, kaupin as ayihnit kau kiwa lik kat bin as dakawi kaunipin yakat kilwi talawak kat, turuh anyang dakni as pa muhnit as sak yakat wiwa bangky, an tanit yakau ahahl singka misin kau pana dakwa bangky an yaka balna dadau. Warangky ban tanitna kau wiwa kat. Kat witingna un awasa bangky misin binini yulni. Al yaka talawak kat witing yaihnit kau pa muhnit as sak yaka tukwak kat putnini sipky. Kauna putwarang kat uk yaka kurah wa rangky, an tanit yakau muih al as simh misin kau wark yamwa bangky an dauwarangky tuniruh balna aka tanitna kau wiwa kat.

Pani muhnit aka tukwa rang sah u dawak ban tanitna kauh wiwarang yah dini wail balna aka?

Stampede push (Contact / Means)


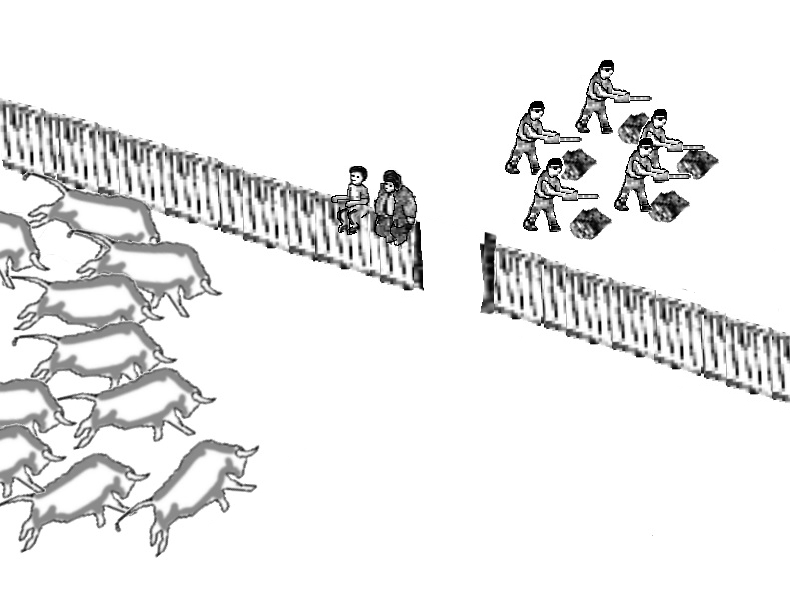


English

You are travelling far away from your community and walking along a fence. You hear a stampede and jump onto the fence next to a large man sitting there. You see that the cattle are headed for an opening in the fence. Beyond this opening is five loggers cutting lumber who will die if the stampede continues its course. They do not know of the stampede due to the noise of the chainsaws and cannot hear the man’s warnings. The only way to keep the five loggers from dying is to push the man on the fence into the opening. In this case, the large man will die but not the loggers.

Would you push the man or let the stampede continue its original course?

Spanish

Usted está viajando lejos de su comunidad y caminando a lo largo de una cerca. Oye una estampida y salta en la cerca al lado de un hombre voluminoso sentado allí. Usted ve que las vacas se dirigen a una abertura en la cerca y más allá de esa hay cinco motosierristas cortando madera, los cuales morirán si la estampida sigue su curso actual. Ellos no saben de la estampida por el ruido de sus motosierras, y además, no pueden oir sus avisos. La única manera de salvar a los cinco motosierristas es empujar a este hombre del cerca en la abertura. En este caso, el hombre voluminoso morirá pero no los motosierristas.

¿Empujaría al hombre o deja la estampida seguir su curso actual?

Mayangna

Muih al as tawan ni kaupak naikau kiwa likky kaupin as yaihnit kau bayak wa likkat bin as dakawi kaupin yakat kilwi muih al salani nuhni yaihnit kau sabatwi talawak turuh anyang dakni as pa muhnit as kau wiwa bangky an tanit yakau talawak ahahl singka misin kau pana dakwa bangky, an yaka balna dadau warangky ban tanitna kau wiwa kat. Kat witingna un awasa bangky misin bin ni yulni. Muihni balna aka daunini awasa kat ta aslahky muih al yaihnit kau sak aka tuk wak pa muhnit yakat buknini kalahwi an alni nuhni aka dauwa rangky, kaput laih singka aka dadau wasa karang.

Alni akat tukwarang yah u dawak ban wiwarang sah dini wail balna aka?

**Study 2 Scenarios**

Scale

After each scenario:

English

What do you think of his decision? That he and one man died and five men did not die.

Spanish

¿Que piensas de su decisión? Que el y un hombre murió y cinco hombres no murieron.

Mayangna

¿Aisa kultamanh alni aka yamna yaka yulni? al aslah daunini yulni, singka daunina awasa yulni.


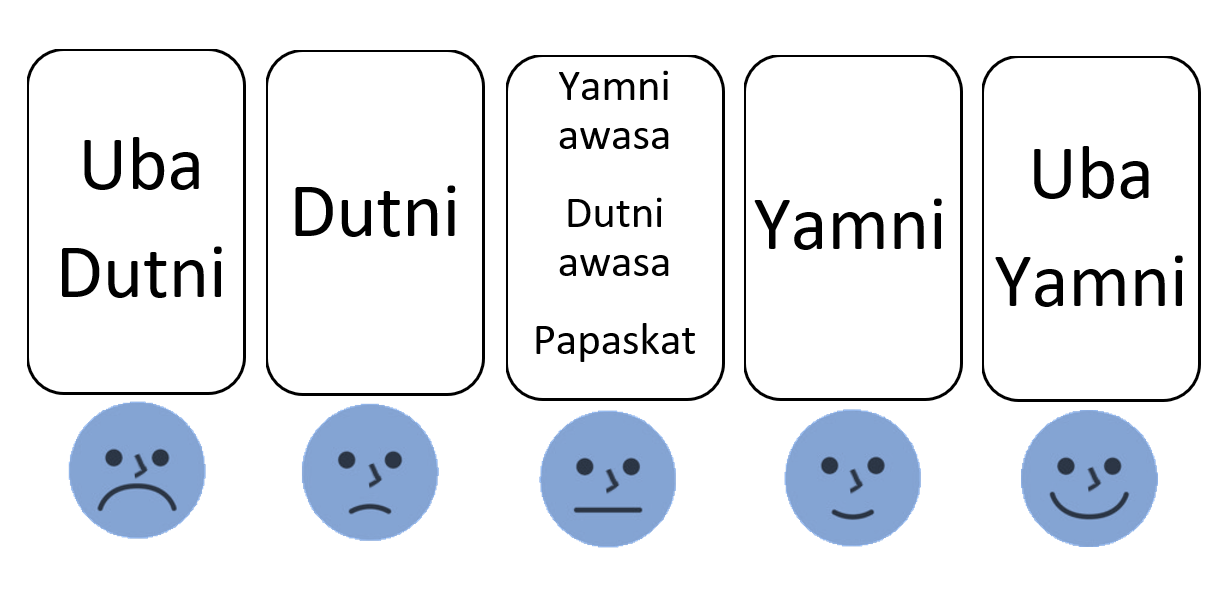


Trolley Switch (No Contact / No Means)


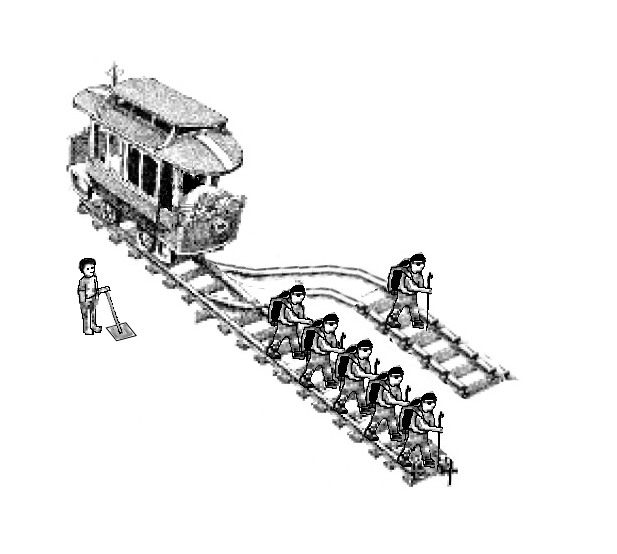


English

An out-of-control train traveling at full speed is headed for five individuals who are on the track and who will die if the train continues its current course. A man is standing next to a lever that can change the train to another track if he activates it. On this track, there is only one person who will die if he activates the lever.

The man decides to activate the lever so that one man is killed and five survive.

Spanish

Un tranvía fuera de control se dirige a toda velocidad hacia cinco personas que se encuentran en la vía, las cuales morirán si el tranvía sigue su curso actual. Un hombre está de pie junto a un mecanismo que encaminará al tranvía por una vía diferente si lo acciona. En esa vía se encuentra una sola persona, la cual morirá si acciona el mecanismo.

El hombre decide accionar el mecanismo para que uno hombre esté matado y los cinco hombres sobreviven.

Mayangna

Tren as brik diska kaiwa sakki, ahahl singka tani pas yakat wiwa tuluky an yaka balna dadau warangky tren ni aka ban kiwa kat. Muih alas yaklauwi talawa witky tren ni akat pana as lawak kat ta uk kau kiunini yulni, an yaka tani kau al as witky an yaka dau warangky kaput yamwa kat.

Alni aka kulwi dakawi yamwa rang kat kaput laih al aslah dau warang an singka dauni awasa yulni.

Trolley Push (Contact / Means)


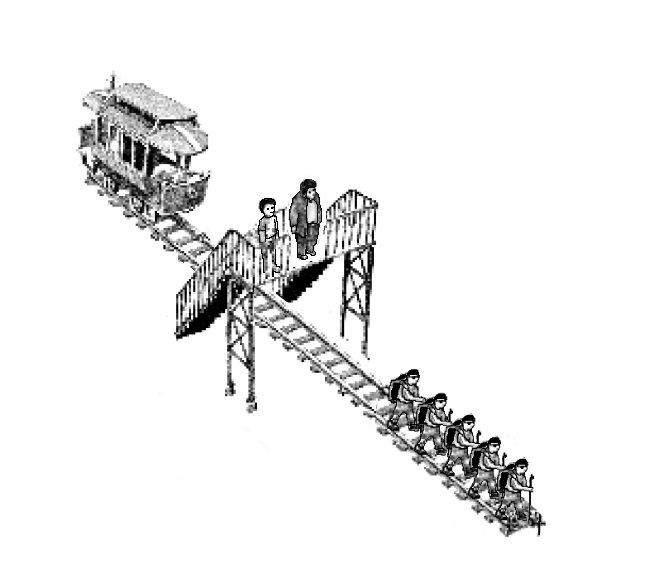


English

An out-of-control train traveling at full speed is headed for five individuals who are on the track and who will die if the train continues its current course. A man is standing next to a large man on a foot bridge over the track. The only way to stop the train so that it does not kill the five individuals is to push the man from the foot bridge into the path of the train. In this case, the large man would die but not the five individuals.

The man decides to push the large man so that one man is killed and the five men survive.

Spanish

Un tranvía fuera de control se dirige a toda velocidad hacia cinco personas que se encuentran en la vía, las cuales morirán si el tranvía sigue su curso actual. Un hombre está de pie junto a un hombre grande en un puente peatonal que está sobre la vía. La única manera de parar el tranvía para que no mate las cinco personas es empujar a este hombre grande del puente peatonal, dentro de la trayectoria del tranvía. En este caso, el hombre grande morirá pero no las cinco personas.

El hombre decide empujar al hombre grande para que uno hombre esté matado y los cinco hombres sobreviven.

Mayangna

Tren as brik diska kaiwa sak ki, ahahl singka tani pas yakat wiwa tuluky an yaka balna dadau wa rangky, tren ni aka ban tanikat kiwakat. Muih al as witky yaihnit kau muih al uk kas nuhni witky dak bayak nini askau. Tren ni aka laih Watnini kat di aslah yamnini ki, al nuhni yaihnit kau wit aka tuknini kalahwi ahahl singka daunini awasa yulni. Kaput laih alni nuhni aka dau warangky.

Alni aka lakwi talawi dawi tuk awarangky aslah daunini yulni singka daunini awasa yulni.

Stampede Gate (No Contact / No Means)


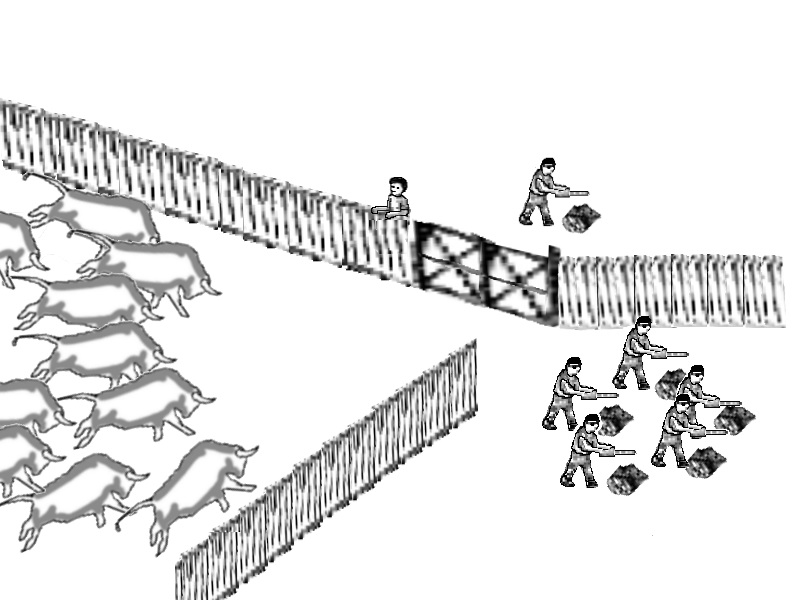


English

A man is travelling far away from his community and decides to rest by sitting on a fence. He sees a stampede of cattle that is headed for an opening in the fence. Beyond this opening is five loggers cutting lumber who will die if the stampede continues its course. They do not know of the stampede due to the noise of the chainsaws and cannot hear the man’s warnings. He sees that there is a gate to his side, and he can push it to close the opening. But if he pushes the gate, another opening will appear on the other side and beyond that opening is a single logger who will die if the stampede continues that course.

The man decides to push the gate so that one man is killed and the five men survive.

Spanish

Un hombre está viajando lejos de su comunidad y caminando a lo largo de una cerca. Oye una estampida y salta en la cerca. El hombre ve que las vacas se dirigen a una abertura en la cerca y más allá de esa hay cinco motosierristas cortando madera, los cuales morirán si la estampida sigue su curso actual. Ellos no saben de la estampida por el ruido de sus motosierras, y además, no pueden oír sus avisos. Ve que hay una puerta que está al lado de él y puede empujarla y cerrar la abertura. Pero si empuja la puerta, otra abertura se abrirá en el otro lado y más allá de esa hay un motosierrista solo, el cual morirá si la estampida sigue ese curso.

El hombre decide empujar la puerta para que uno hombre esté matado y los cinco hombres sobreviven.

Mayangna

Muih al as tawan ni kaupak kalahna likky, kaupin as yahnit kau kiwa likkat bin as daka wi kauni pin yakat kilwi talawak kat, turuh anyang dak ni as pamuhnit as kau wiwa bangky, an tanit yakau ahahl singka misin kau pana dakara bangky, an yaka balna dadau warangky ban tanitna kau wiwa kat. Witingna un awasa bangkymisin bin ni yulni. Al yaka talawak kat witing yahnit kau pa muhnit as sak yakatukwak putnini sipky. Kauna putwarang kat pamahnit uk yaka kurah warangky an tanit yakau muih al as simh misin kau wark yamwa sakky an dauwaranky tuniruh balna aka tanitna kau wiwa kat.

Alni aka lakwi talawa dawi tokwa rangky pa muhnit yahnit kau sakyaka al aslah daunini yulni an singa dauninina awasa yulni.

Stampede push (Contact / Means)


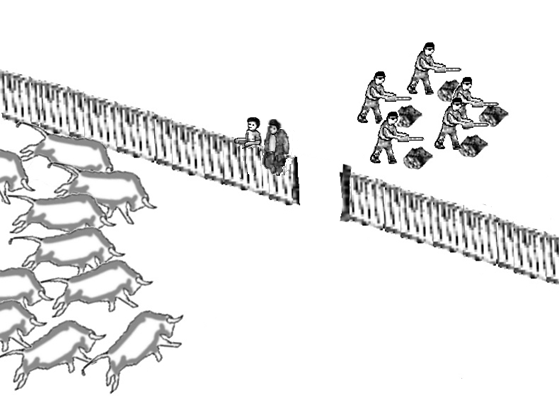


English

A man is travelling far away from his community and walking along a fence. He hears a stampede and jumps onto the fence alongside a large man sitting there. The man sees a stampede of cattle that is headed for an opening in the fence. Beyond this opening is five loggers cutting lumber who will die if the stampede continues its course. They do not know of the stampede due to the noise of the chainsaws and cannot hear the man’s warnings. The only way for the five loggers to not die is if the man pushes the other man from the fence into the opening. In this case, the large will die but the stampede will stop and the loggers will survive.

The man decides to push the large man so that one man is killed and the five men survive.

Spanish

Un hombre está viajando lejos de su comunidad y caminando a lo largo de una cerca. Oye una estampida y salta en la cerca al lado de un hombre grande sentado allí. El hombre ve que las vacas se dirigen a una abertura en la cerca y más allá de esa hay cinco motosierristas cortando madera, los cuales morirán si la estampida sigue su curso actual. Ellos no saben de la estampida por el ruido de sus motosierras, y además, no pueden oir sus avisos. La única manera para que los cinco motosierristas no mueren es si el hombre empuja a este hombre del cerca en la abertura. En este caso, el hombre grande morirá pero la estampida parará y los motosierristas sobrevivirán.

El hombre decide empujar al hombre grande para que uno hombre esté matado y los cinco hombres sobreviven.

Mayangna

Muih as tawani kaupak naikau kiwa likky kaupin as yaihnit kau bayakwa likkat gin as dakawi, kaupin yakat kilwi muih al salani nuhni yaihnit kau sabatwi talawak turuh anyang dakni as pamuhnit as kau wiwa bang ky an tanit yakau talawak ahahl singka misin kau pana dakwa bangky, an yaka balna dadau waranky ban tanitna kau wiwa kat. Kat witingna un awasa bangky misin bin ni yulni.

Muihni balan aka daunini awasa kat ta aslahky miuh al yaihnit kau sak aka tuk wak pa muhnit yakat buknini kalahwi an alni nuhni aka dauwa rangky, kaupt laih singka aka dadau wasa rarang.

Alni aka kulwi dakawi dawri alni nuhni akat tuk warang aslah daunini yulni singka daunini awasa yulni.

Truck Silent (No Action)


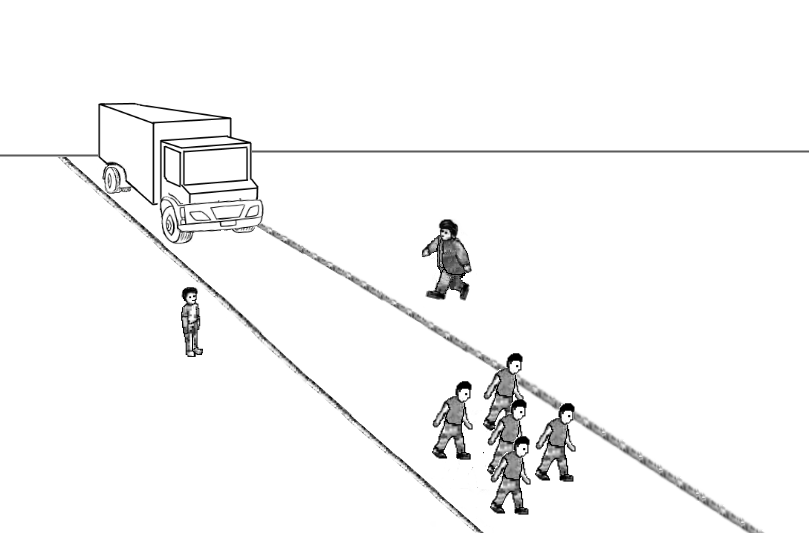


English

A man is standing near the side of the road when he sees a truck speeding along. It is headed towards a group of five men, who do not hear or see it, and if nothing is done, it will certainly hit and kill them.

Across the road is another man who is about to cross the road. If he walks into the road, the truck will certainly hit him and kill him. But if this occurs, the truck will stop and will not continue on toward the five men, and they will not die.

If the man on this side of the road shouts at the other man as says “watch out!”, the man will definitely not walk into the road into the path of the truck. However, the truck will continue toward the five men and will kill them. If the man says nothing, the truck will kill the man crossing the road.

The man decides to no say anything so that one man is killed and the five men survive.

Spanish

Un hombre está cerca de la orilla del camino cuando él ve un camión que viene muy rápido. Está dirigido a un grupo de cinco hombres que no oyen ni ven el camión, y si nada pasa, el camión por cierto les golpeará y les matará.

En otro lado del camino hay otro hombre que está por cruzar el camino. Si él camina en el camino, por cierto, el camión le goleará y le matará. Pero si esto sucede, el camión parará y no continuará y los cinco hombres y ellos no morirán.

Si el hombre que está en la orilla del camino grita al otro hombre y dice “!Cuidado!”, el hombre no andará en el camino en la trayectoria del camión y estará seguro. Pero el camión continuará hacia los cinco hombres, y les matará. Si el hombre dice nada, el camión continuará y matará el hombre que cruza el camino.

El hombre decide que no va a decir nada para que uno hombre esté matado y los cinco hombres sobreviven.

Mayangna

Muih al as truk kira tani yaihnit kau witki dawak, talawak truk as sirihni palni kaiwa sakki muih ahahl singka wiwa tulu tani yakat, muihni balna yaka daka kawasa an talawasa wiwa tuluky bahang di iwarangky.

Muih al as ta yaihnit kau wit aka kalyulwang uk akat “aman talah!” kalatangkat dakwasa karangky truk tani yakat, kauna truk ni aka ban kaiwi muih ahahl singka akt imuk waranky. As yaka amantalah yulwasakat truk ni aka ahal singka aka imok waranky.

Al ni aka kulwi dakawi di as yulwasa krangky al aslah daunini yulni singka daunini awasa yulni

Truck Call (Action)

.


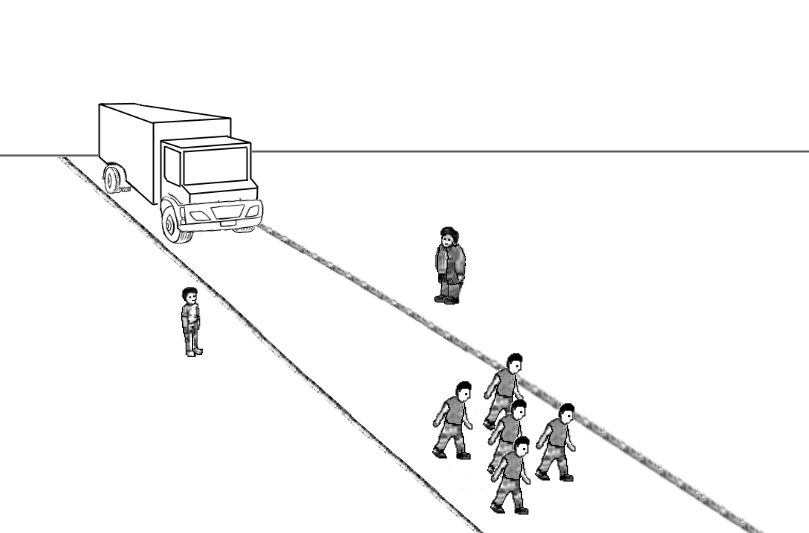


English

A man is standing near the side of the road when he sees a truck speeding along. It is headed towards a group of five men, who do not hear or see it, and if nothing is done, it will certainly hit and kill them.

Across the road is another man. If the man on this side calls out to the man and says ‘come here!’ the man will walk into the road in the path of the truck, the truck will hit him and kill him. But if this occurs, the truck will stop and will not continue on toward the five men, and they will not die.

If the man does not say anything, the truck will continue on and kill the five men.

The man decides to call out to the other man so that he comes and one man is killed and the five men survive.

Spanish

Un hombre está cerca de la orilla del camino cuando él ve un camión que viene muy rápido. Está dirigido a un grupo de cinco hombres que no oyen ni ven el camión, y si nada pasa, el camión por cierto les golpeará y les matará.

En otro lado del camino hay otro hombre. Si el hombre en este lado llama al hombre y dice “!ven aca!”, el hombre andará en el camino en la trayectoria del camión, y el camión le golpeará y le matará. Pero si esto sucede, el camión parará y no continuará hacia los cinco hombres y ellos no morirán.

Si el hombre no dice nada, el camión continuará y matará los cinco.

El hombre decide llamar al hombre para que venga y uno hombre es matado y los cinco hombres sobreviven.

Mayangna

Muih al as truk kira tani yaihnit kau witki dawak talawak truk as sirihni palni kaiwa sakki muih ah ahl singka wiwa tulu tani yakat, muihni balna yaka daka kawasa an talalawa sa wiwa tuluky bahang di iwarangky.

Dawak nahas kau muih al as talna witki witing yaklauwi wauwarangky as yak “akau aiwah!” dak kaiwa sakkat trukni aka taihwi iwaranky. Kaput laih truk ni aka watwa rangky dawak muih ahahl singka balna aka dadowasa kranky.

Alni aka di as yamwasa kata, singka pak daduwarangky.

Alni aka wauwak dakwarang kat al aslah dauwarang singka awasa.

Stampede Silent (No Action)


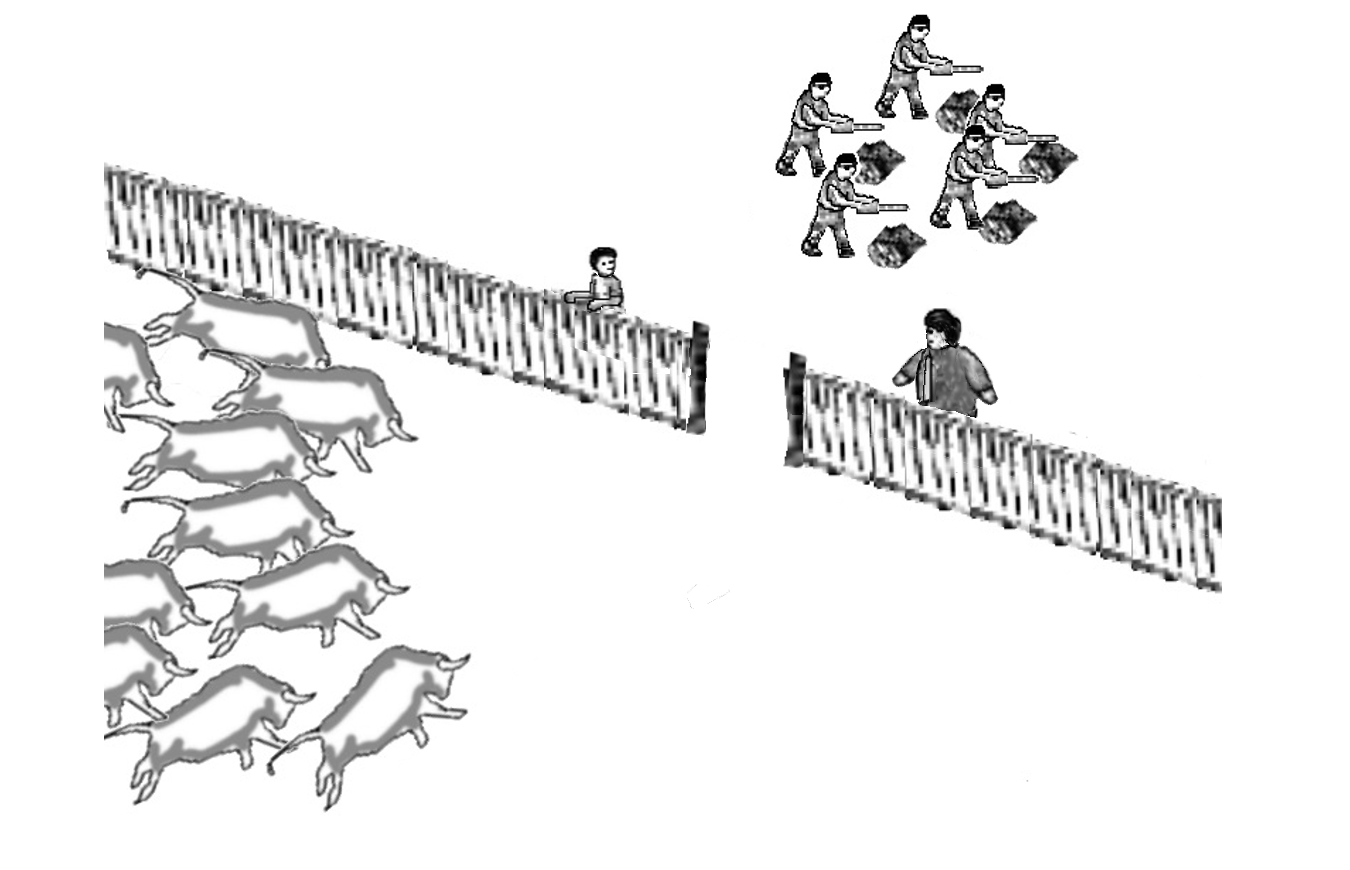


English

A man is travelling far away from his community and decides to rest by sitting on a fence. He sees a stampede of cattle that is headed for an opening in the fence. Beyond this opening is five loggers cutting lumber who will die if the stampede continues its course. The loggers do not know of the stampede due to the noise of the chainsaws and cannot hear the man’s warnings.

There is also a man walking toward the opening in the fence. If he continues on this path, the cattle will kill him, but if this happens, the stampede will stop, saving the lives of the five loggers.

If the man sitting on the fence yells at the man “watch out!”, the man will stop from walking into the path of the stampede and will be safe. However, the cattle will continue toward the five loggers, killing them.

The man decides to not say anything so that the man who is walking is killed and the five loggers survive.

Spanish

Un hombre está viajando lejos de su comunidad y decide descansar y se siente encima de un cerco. Él ve una estampida que está dirigida a una abertura en el cerco. Más allá de esa hay cinco motosierristas cortando madera, los cuales morirán si la estampida sigue su curso actual. Ellos no saben de la estampida por el ruido de sus motosierras, y además, no pueden oir sus avisos.

También hay un hombre quien anda hacia la abertura en el cerco. Si él continua, por cierto, las vacas le matarán, pero si esta sucede, la estampida parará, y los cinco motosierristas no morirán.

Si el hombre sentado en el cerco grita al hombre y dice “!Cuidado!”, el hombre no andará a la abertura en la trayectoria de la estampida y estará seguro. Pero las vacas continuarán hacia los cinco motosierristas y les matarán.

El hombre decide que no va decir nada para que uno hombre esté matado y los cinco hombres sobreviven.

Mayangna

Muih al as tawan ni kaupak naika kalahna lik ky minit as ris dunini yulni, kau pin as makau kilwi sabat wi win yaklawi talawak turuh anyang dakni as aiwa bangky, dawak tanit kau talawak muih ahahl singka misin kau pana dakwa bangky, tuniruh balna aka tanitna kau wiwa kat ahahl ni balna aka dadau wa rangky, kat dakawasa bangky bin ni yaka yulni.

Muih al uk asbik kaiwa likky simh turuh aiwa tani yaka tan dauwarang ky, kauna kaput kalah warang kat tuniruh balna aka wawat warangky an ahahl singka misin kau wark yamwa bang yaka dadau wasa karangky.

Muih al kaupin kau sabatna sak daika wauwarang “amantalah!” atarang kat mihni al as yaka un kalah wi dakwasa karang an dauwasa karanky, kauna tuniruh balna aka tanitna kau wiwi muih ahahl singka aka imuk warang.

Alni as aka kulwi dakawi di as yulwasa karang al aslah daunini yulni singka daunini awasa yulni.

Stampede Call (Action)


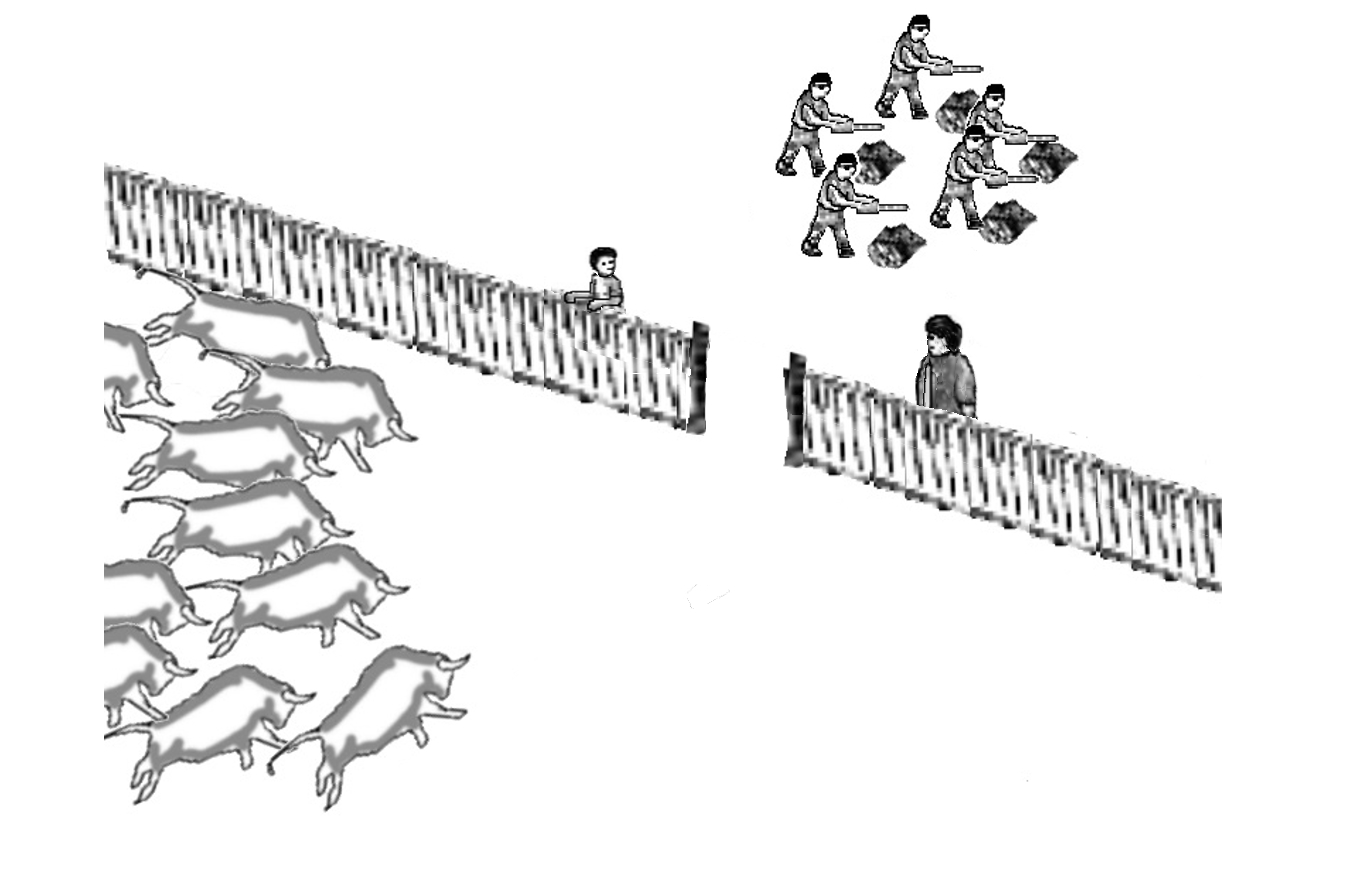
 English

A man is travelling far away from his community and decides to rest by sitting on a fence. He sees a stampede of cattle that is headed for an opening in the fence. Beyond this opening is five loggers cutting lumber who will die if the stampede continues its course. The loggers do not know of the stampede due to the noise of the chainsaws and cannot hear the man’s warnings.

There is also a man standing on the other side of opening in the fence. He doesn’t know of the stampede. If the man sitting on the fence calls to the other man and says “come here!”, the man will walk toward the opening and the cattle will kill him. But if this happens, the stampede will stop, and the five loggers will not die.

If the man on the fence doesn’t say anything, the cattle will continue and kill the five loggers.

The man decides to call out to the other man so that he comes and one man is killed and the five men survive.

Spanish

Un hombre está viajando lejos de su comunidad y decide descansar y se siente encima de un cerco. Él ve una estampida que está dirigida a una abertura en el cerco. Más allá de esa hay cinco motosierristas cortando madera, los cuales morirán si la estampida sigue su curso actual. Ellos no saben de la estampida por el ruido de sus motosierras, y además, no pueden oir sus avisos.

También hay un hombre quien está parado en otro lado de la abertura en el cerco. Él no sabe de la estampida. Si el hombre en el cerco llama al hombre y dice “!ven aca!”, el hombre andará hacia la abertura y las vacas le matarán. Pero si esto sucede, la estampida parará, y los cinco motosierristas no morirán.

Si el hombre en el cerco no dice nada, las vacas continuarán y matarán los cinco motosierristas.

El hombre decide llamar al hombre para que venga y uno hombre esté matado y los cinco motosierristas sobreviven.

Mayangna

Muih al as tawan ni kaupak naikau kalahna likky, minit as ris dunini yulni kaupin as kau kilwi sabat wi wiri yaklawi talawak turuh anyang dakni as ailwa bangky, dawak tanit kau talawak muih ahahl singka misin kau pana dakwa bangky, dawak tuniruh balna aka tanitna kau wiwa kat ahahlni balna aka dadau rangky kat dakawasa bangky misin bin ni yaka yulni.

Muih al uk asbik witky kaupin yaihnit kau simh turuh aiwa bang tani yakat, muih alukbik kaiwa likky tanit yakau an un awasaky, turuh aiwa bang yak al as talna wit aka yulwarang “akau aiwah!” kal yulwarang kat pa muhnit akat kaiwak tuniruh balna aka yaklauwi taihwi iwarang al akat. Kaput bayakwa kat tuniruh balna aka wawatwa rangky an ahahl singka misin kau wark yamwa bang aka dadauwasa karang.

Muih al kaupin kau sak aka días yulwasa kat muih ahahl singka aka dadau warang.

Alni as aka kulwi dakawi wauwak kaiwarang, muih al aslah daunini yulni, singka aka daunina awasa yulni.

1. Wiegmann A, Okan Y, Nagel J. Order effects in moral judgment. Journal of Philosophical Psychology. 2012;25(6):813-36.
