# Supplementary material for: Small-scale utilitarianism: High acceptance of utilitarian solutions to Trolley Problems among a horticultural population in Nicaragua
Source: PLoS One. 2021 Apr 5;16(4):e0249345. doi: 10.1371/journal.pone.0249345 (PMC8021155; doi:10.1371/journal.pone.0249345)
Supplement: Attachment — Submitted filename: Winking-Koster 2020 Response to Reviewers 02.02.21.docx [file pone.0249345.s002.docx]

Dear editor:

We thank the two reviewers for their thoughtful comments. We have tried to accommodate all of their suggestions and believe the paper to be much improved. Major additions include a more thorough exploration of the theoretical foundations for the study in the introduction as well as new multivariate analyses of Study 1 and Study 2 results. We provide detailed descriptions of the revisions below:

**Reviewer 1:**

**Recommendation**: “First, I recommend that the authors explicitly explain these biases in more detail on p. 3 so that a more general audience reader understands the implications of these biases in decision making in these types of scenarios.”

**Revision**: Additional language was added to the second paragraph of the Introduction (lines 54-63), and now reads:

“Previous research has identified three elements in this footbridge variant that reduce perceptions of permissibility. First, individuals tend to judge actions that involve physical touch as less permissible (*contact bias*) (5, 6). Second, scenarios are deemed less permissible when they require another individual to serve as a means to the end (i.e., the large man is *used* to stop the Trolley) (*means bias*) (4, 7). Finally, the third factor, which is evident in both the traditional and the footbridge scenario, is the fact that the bystander must *act* in order to bring about the utilitarian solution, as opposed to simply allowing for the utilitarian solution to come about naturally (*omission bias*) (8-11). For instance, it would presumably be less offensive if the bystander simply refrained from warning the large man who was distracted and was about to walk off the footbridge and inadvertently stop the trolley from killing five men.”

**Recommendation**: “I recommend that the Discussion section starts with a more general introductory sentence(s) and refer to the aims of the study. Then proceed to address those aims in a systematic manner to ensure that it is clearer and more reader-friendly or easy to follow.”

**Revision**: We have added a paragraph to the beginning of the discussion restating the objectives of the study (Lines 467-474), and we have added more sign post language throughout (e.g., Lines 475 and 494).

**Reviewer 2:**

**Recommendation**: “The introduction lacks a clear rationale for the study other than simply running a study in a novel population (which is acceptable but could be better). A bit more about why variability in moral responding is an issue, and what it means for moral psychology would strengthen the introduction a bit. The dual process framework could be an avenue: it expects higher order cognition to drive U responding, and intuition to drive D responding. Little education would predict more D responses in societies with less formal education.”

**Revision**: We have created a new section titled “Moral Reasoning and Small-scale Society”, which also incorporates some of the existing language (Lines 79-149). In it we explore three theoretical approaches: 1) the fact that Dual Process model might predict more deontologically-driven reasoning, 2) that the literature on relatedness effects might predict more utilitarian-driven reasoning, and 3) that ad hoc sociocultural factors need to be explored, as they might swamp other patterns.

**Recommendation**: “It was not clear why the trolley vs stampede scenarios were used instead of only the culturally adapted versions. The authors acknowledge that their participants had problems understanding the trolley versions. Moreover, the potential difference test between trolley vs stampede versions is not reported.”

**Revision**: We now report the effect of using the culturally adapted version in Tables 1 and 3, using a mixed-effects logistic and linear regression respectively, and report no significant effect. We explore this in the text in Lines 367-378 and 397-399.

**Recommendation**: “Next, compared to the older preprint (which I had the opportunity to read few months ago on psyarxiv) includes the ordering effect analysis. It shows that being responded first has an effect on the likelihood of the utilitarian response (not clear how it was estimated in the SEM, since the DV is binary, and the B is > 1!; I assume it is an error).”

**Revision**: The analysis now exists in the Supporting Information (Table S1). We’re not entirely sure of the possible problem here. We used a multilevel probit analysis, for which the coefficients represent z-score changes along the inverse normal distribution, and for which a B>1 is possible. We also now include an examination of just respondent’s first round (Table S2) in keeping with changes made to Study 2 (see below) and indicate that the difference between the Trolley Scenarios becomes more apparent (p=0.097) (Lines 388-391).

**Recommendation**: “The real issue with ordering effects is more complex. Alex Wiegmann showed multiply times that if the order is indirect -> direct dilemma, respondents show no ordering effect. Yet, having responded to direct dilemma first, participants better realize their action is causing the death of one person, and therefore tend to be less utilitarian is subsequent indirect dilemma. So, it is not enough to look at the relative position of a dilemma, but also at the type of preceding dilemma. Maybe an LME with order, dilemma type (trolley vs stampede), dilemma version (direct vs indirect) would provide more direct answers.”

**Revision**: We now include in the Supporting Information an analysis of the effect of having responded to a push scenario (direct) on later responding to a switch/gate scenario (indirect), as well as the reverse (Tables S3a/b). No order effects were detected, although sample sizes were very low after limiting the test to those situations.

**Recommendation**: “Next, gender effects can be of interest here. I looked at the raw data provided by the authors, and saw that female are far less utilitarian than males. Maybe it would be helpful to additionally control for sex effects in the test above? These can contaminate the main analysis, assuming genders are not perfectly distributed across conditions.

**Revision**: For both studies, we now incorporate mixed-effects models to control for the effects of age and gender (and also test for the effects of the culturally adapted vs traditional versions). These are reported in Table 1 for Study 1 and Table 3 for Study 2. Men were found to be significantly more likely to choose the utilitarian solution in Study 1, but no effect was found in Study 2. Age is a significant predictor in the Stampede scenarios in Study 1. We report these in the text in Lines 367-378 and 397-399.

**Recommendation**: “Again, the ordering effects are real problem, and can severely affect the responding pattern. Could you supplement the main analysis with one that tests only the first trials for each participant?”

**Revision**: We now include an analysis for Study 2 that only uses individuals’ responses to the first scenario the responded to (Table S4d). The overall interpretation of the results remain unchanged. The pair with the smallest effect (Truck Silent/Truck Call, d=0.08) switched directions, but the effect remains negligible.

**Recommendation:** Finally, Bostyn in Psych Sci 2018 and PB&R 2019 used such formulation of question to only investigate utilitarian inclinations, claiming that the answer to a Q judging an agent that acted deontologically to test D inclinations. My five, yet unpublished experiments fully support this idea. So, Study 2 tested utilitarian preferences only.

**Revision**: We state this explicitly in the methods section (Lines 280-282), which now reads:

“Following Arbarbenell and Hauser (9), this study only evaluates ratings of utilitarian decisions, and so it does not speak to perceptions of deontological decisions.”

**Recommendation**: Again, the analysis would be much more sensitive if it would adopt LME modelling instead od series of independent Chi-2 tests. The study would also benefit from clear explanation why for each dilemma, call vs push version was used (to test for the contact vs means bias), and a direct statistical test of this bias. Experiment 2 would also benefit from a figure illustrating its results.

**Revision:** We now included mixed-effects analyses of Study 1 (Table 1) and Study 2 (Table 3) as well as paragraphs explaining the results (Lines 365-370 and 395-397). We are not clear on the second point, but the biases are now tested with both bivariate and multivariate tests. Finally, we now include Figure 3, which presents the means ratings across the 8 scenarios in Study 2.

**Minor Recommendations:**

Line 287: a part of a sentence is repeated, “and a power of 0.9 to detect a medium effect size of Cohen’s d=0.6”.

-The language was in fact slightly different. We use d=0.6 as a standard for a medium effect size, and we therefore included both an empirically derived effect size and a standard medium effect size for both scenarios. But we agree that it causes confusion and adds little, so we deleted it (Line 326).

Line 315: Although the Trolley scenarios only reveal a suggestive effect (Trolley Switch: 93.5%, Trolley Push: 78.1%, Fisher’s Exact, p=0.148). The effect is either there or not. I don’t know what an suggestive effect with p = > .10 is in statistical terms.

-We have changed the language to read, “The effect exhibited in the Trolley scenarios is in the predicted direction but is not significant…”

Line 344-346: The authors write: In a robustness check, we examined the possibility of order effects, and although this supplementary analysis provided evidence for a moderate effect, the inferences for the comparisons between the scenarios remain consistent (Electronical Supplemental Materials, Tables S2a–d). I don’t see how ESM supports this claim, please elaborate.

-We have changed the language so that Lines 410-417 now read:

“In a robustness check, we examined the possibility of order effects (Supplemental Materials, Tables S4a-e). This supplementary analysis revealed a moderate effect with individuals reporting lower ratings for the first scenario. However, the inferences for the comparisons between the scenarios remain consistent. The most substantive change was that there the non-significant effect between Stampede Gate and Stampede Push (Table 2, p=0.193) becomes more pronounced in a mixed-effects regression controlling for whether or not scenarios were presented in the first position (Stampede Gate, EM Mean=3.64, Stampede Push, EM Mean=3.40; p=0.085) (Supporting Information, Table S4e).”
